# Evidence of trans-generational developmental modifications induced by simulated heat waves in an arthropod

**DOI:** 10.1038/s41598-020-61040-z

**Published:** 2020-03-05

**Authors:** A. Walzer, H. Formayer, M.-S. Tixier

**Affiliations:** 1 Division of Plant Protection, Department of Crop Sciences, University of Natural Resources and Life Sciences, 1180 Vienna, Austria; 2 Institute of Meteorology, Department of Water, Atmosphere and Environment, University of Natural Resources and Life Sciences, 1180 Vienna, Austria; 3 CBGP, Montpellier SupAgro, INRA, CIRAD, IRD, Univ. Montpellier, Campus International de Baillarguet, CS 30016 34988 Montferrier-sur-Lez, cedex Montpellier, France

**Keywords:** Developmental biology, Ecology, Evolution, Climate sciences

## Abstract

Heat waves are considered to pose a greater risk to arthropods with their limited thermoregulation abilities than the increase of mean temperatures. Theoretically, within- and trans-generational modifications may allow populations to keep pace with rapidly occurring heat waves. Here, we evaluated this assumption using individuals of predatory mite *Amblydromalus limonicus* from the F1 and F2 generation, which were exposed to summer or simulated heat wave conditions during juvenile development. Independent of generation, survival and male body size were insensitive to heat waves. Heat stress elongated juvenile development of F1 males and females, and lowered the F1 female size at maturity indicating non-adaptive within-generational effects. Trans-generational modifications speeded up the development of F2 males and females and resulted in larger body size of F2 females deriving from the heat wave-experienced F1 generation. Faster F2 development should be adaptive, because it reduces the exposure time to heat waves and promotes an early beginning of mating activities. Being large at extreme high temperatures maybe a benefit for the F2 females, because large individuals are less vulnerable to dehydration and overheating. Thus, the potential fitness loss from reduced F1 growth should be compensated by increased fitness in the F2 indicating adaptive trans-generational modifications.

## Introduction

Climate warming has wide-ranging impact on organisms limited in their internal thermo-regulation such as arthropods, because temperature nearly affects all vital processes such as survival, development and reproduction^1^. Arthropods may respond to changing thermal conditions via behavioural thermoregulation reflected in range shifts, genetic adaptation, and phenotypic plasticity^2^. Behavioural responses by large-range avoidance and the selection of thermally suitable locations resulting in poleward and upward range shifts are well documented in some taxa^3^, but should be reserved for arthropods with well-developed dispersal abilities such as some butterflies, dragonflies and grasshoppers^4^. The opportunities of the majority of arthropods, however, are limited to genetic modifications and/or phenotypic plasticity^5,6^, whereas the former mechanism might be appropriate to adopt to long-term environmental changes such as the annual increase of the mean temperatures^7^.

Heat waves, however, are fundamentally different from mild warming in the ecological consequences for arthropods and their evolutionary responses^8–10^. First, heat waves can exceed the critical thermal maxima of arthropods within few hours. Accordingly, even when the daily mean temperatures are still within a suitable thermal range, a single aberration beyond their critical thermal maxima can be fatal^11^. Consequently, these rapid thermal changes are considered to have stronger effects on survival, development and reproduction of arthropods than small shifts in mean temperatures^12,13^. Second, the time frame for effective genetic adaptations, however, is significantly shorter for populations exposed to rapidly occurring heat waves than to the slow increase of the mean temperatures, which should make plastic modifications more likely^8,9^.

Temperature is one of the most prominent agents of developmental plasticity^14^, which may result in adaptive modifications to cope with heat waves. Such adaptations usually occur within a generation (i.e. within-generational plasticity (WGP)]. Heat wave-experienced parents, however, may also modify age and size of the next generation [i.e. trans-generational plasticity (TGP)]^15^. Ultimately, thermal TGP may allow populations with short generation times to keep pace with rapidly changing thermal conditions under heat waves by phenotypic modifications in a temporally adequate manner^8,16^. Few studies addressed heat wave effects on the parental and offspring development, which provided diverging results on the potential adaptive values of WGP and TGP. For example, exposure to heat waves resulted in costs for mothers (reduced fecundity) and their offspring (lower birth weight) in the grain aphid *Sitobion avenae* F.^17^. Trans-generational modifications in the spider mite *Tetranychus urticae* resulted in maladaptive offspring effects (longer juvenile development) under permanent heat stress^18^. Plastic modifications to heat shocks also reduced maternal fecundity, but increased the offspring heat resistance in the springtail *Orchesella cincta* L.^19^.

We studied the WGP and TGP effects induced by heat waves on life history traits of the plant-inhabiting predatory mite *Amblydromalus limonicus* Garman & McGregor (Acari: Phytoseiidae). This mite occurs naturally in subtropical and temperate climate zones with spatiotemporal heterogeneous environments located in North-, Central- and South-America, Australia and New Zealand^20^, indicating that *A. limonicus* is selected for high thermal plasticity.

Predatory mites of the family Phytoseiidae are ideal objects to study heat wave-induced within- and trans-generational effects for several reasons. First, these plant-inhabiting mites are tiny and wingless (body length: 300–600 µm^21^), which strongly limits the abilities of the mites to cover large distances by locomotion^22^. Thus, the predatory mites are usually forced to cope *in-situ* with heat waves. Second, predatory mites have the potential to adjust to changed environmental conditions by means of plastic modifications. WGP effects in predatory mites are well documented as responses to food shortage^23–25^, heat stress^26^, and predation risk^27,28^. TGP effects were observed under food stress affecting offspring body size, whereas the exposure period influenced the sex-specific phenotypic shifts in size. Only females from the second generation (F2) were small, when their mothers from the first generation (F1) were food-stressed during their whole juvenile developmental period^24^. Contrary, F2 females were large, when deriving from F1 mothers, which were food-stressed during the reproductive phase^25^. Finally, the matching of F1 and F2 environment is considered to be a prerequisite for adaptive TGP effects on offspring traits^13,29^. Hence the fast juvenile development of predatory mites within few days^23^ clearly increases the probability that the F1 environmental conditions correlate with the F2 environmental conditions during a heat wave. Consequently, the objectives of this study were to evaluate the heat wave effects on survival, age and size at maturity of both F1 and F2 generation of *A. limonicus*. We hypothesized that (1) the F1 generation reacts to heat waves by plastic responses related to age and size at maturity (WGP), (2) the F1 thermal environment also influences the life-history traits of the F2 generation (TGP), and (3) the WGP and TGP effects are sex-specific in these mites with a female-biased size dimorphism^23^, i.e. the larger females are more strongly affected by TGP than the smaller males.

## Methods

### Species sampling and identification

Specimens of *A. limonicus* were sampled in 2014 on apple trees in La Tallada d’Emporda, near Girona, Spain, which were used to establish the lab population. The predatory mites were identified as *A. limonicus* by using the identification key of Schuster & Pritchard^21^ (1963) based on morphological characters. To confirm the morphological diagnosis, a molecular identification was conducted using the 12S rRNA marker, largely used for diagnostic issues in the family Phytoseiidae^30^. Eight specimens collected in Spain and three specimens commercialized by Koppert NL were analyzed. Total genomic DNA was individually extracted from the 11 females, using a Qiagen DNeasy tissue kit (Qiagen, Hilden, Germany), according to the DNA extraction protocol^31^. After DNA extraction, PCR was conducted using the primers to amplify the DNA fragment 12S rRNA^32^. Electrophoresis was carried out on a 1.5% agarose gel in 0.5 X TBE buffer during 20 min at 135 volts. PCR products were sequenced along both strands using Dynamic ET Terminator Cycle Sequencing kit, and purified using ExoSAP-IT (Amersham*)*. The sequencer used was the Megabase 1,000 apparatus. The DNA sequences were blasted in the Genbank database to determine, if the specimens considered correspond to Phytoseiidae. Molecular analyses were conducted using Mega 6.0.6^33^. The distance matrices were elaborated using the Kimura 2-parameter model.

Molecular identification revealed that the genetic distances between the eight specimens collected in Spain were equal to 0%, showing no differences for the 12S rRNA fragment among the specimens considered. These specimens differ from those commercialized by Koppert by a low mean distance of 0.05% (min: 0%; max: 1.7%), which was within the intraspecific variation usually observed for Phytoseiidae^34^. The specimens herein considered belong thus to the species *A. limonicus*. The accession numbers of these sequences in Genbank are: (i) population from Spain: SUB5946808, seq. 4 to 11 (MN180274-MN180281), (ii) commercial population from Koppert: SUB5946808, seq1 to 3 (MN180271-MN180273).

### The temperature regime in the field

Climatic data from the sampling location of *A. limonicus* in Northeastern Spain (La Tallada d’Emporda, Girona, Spain; 42.0541°N, 3.0614°E) were generated for the summer periods 2007 to 2017 (June, July, August, September) from the high-resolution gridded data set of daily climate over Europe^35^. The daily maximum summer temperatures (T_max_) over eleven years were used to determine the number and duration of heat waves at the location, where *A. limonicus* is well established in the field. A heat wave was defined as the longest period of consecutive days satisfying the following three conditions: (i) T_max_ must be >30 °C for the first three consecutive days, (ii) the daily T_max_ can be below 30 °C, but not 25 °C, for single days during a heat wave period and (iii) the average T_max_ is >30 °C for the entire period^36^.

The thermal data evaluation revealed that the field population of *A. limonicus* in La Tallada d’Emporda was exposed to 44 heat waves with a duration ranging from 4 to 44 days between 2007 and 2017. The statistical values were: mean duration = 11.9 days, median duration = 8.0 days, 0.25 quartile = 6.0 days, 0.75 quartile = 14.0 days. The juvenile development of *A. limonicus* is temperature-dependent and can last from 6 to 8 days^37^, which makes the thermal matching of the F1 and F2 environment during a heat wave likely at least when young F1 parents begin with mating and egg production at the beginning of a heat wave.

### Determination of the diurnal temperature fluctuations

The chosen diurnal temperature fluctuations should simulate heat wave and summer conditions^36^. The temperature regimes were sub-lethal for the individuals allowing egg hatching and juvenile development to rule out selective mortality effects^15^. The specific thermal values were: (1) heat wave conditions, daily maximum temperature (T_max_) = 35 °C, daily minimum temperature (T_min_) = 20 °C, daily mean temperature (T_mean_) = 26.7 °C, and (2) summer conditions: T_max_ = 30 °C; T_min_ = 15 °C; and T_mean_ = 21.7 °C (Table 1). The relative humidity in both temperature treatments was kept constant at 60 ± 5%.

**Table 1:** Thermal conditions for the summer and heat wave treatments used.

Time of the day (h)	Temperature (°C)	
	Summer conditions	Heat wave conditions
09:00–12:00	25	30
12:00–14:00	30	35
14:00–17:00	25	30
17:00–23:00	20	25
23:00–01:00	15	20
01:00–09:00	20	25
Mean temperature (°C)	21.7	26.7

Light conditions corresponded to long-day conditions (L:D = 16 h:8 h). Relative humidity was kept constant at 60 ± 5%.

### Rearing and experimental units

80–100 *A. limonicus* individuals from the sampling location (La Tallada d’Emporda, Girona, Spain) were used to initiate the lab population. Acrylic plates placed on water-saturated foam cubes in plastic boxes half-filled with water served as rearing arenas. Eggs and mobile stages of *Tetranychus urticae* (Acari: Tetranychidae) and pollen of narrow-leaved cattail *Typha angustifolia* (Nutrimite, Biobest N.V., Westerlo, Belgium) were provided in regular intervals as food resources for the predators (see^38^ for details). All rearing units were placed in climate chambers at 25 ± 1 °C, 60 ± 10% RH and 16:8 h L:D.

**Figure 1:**
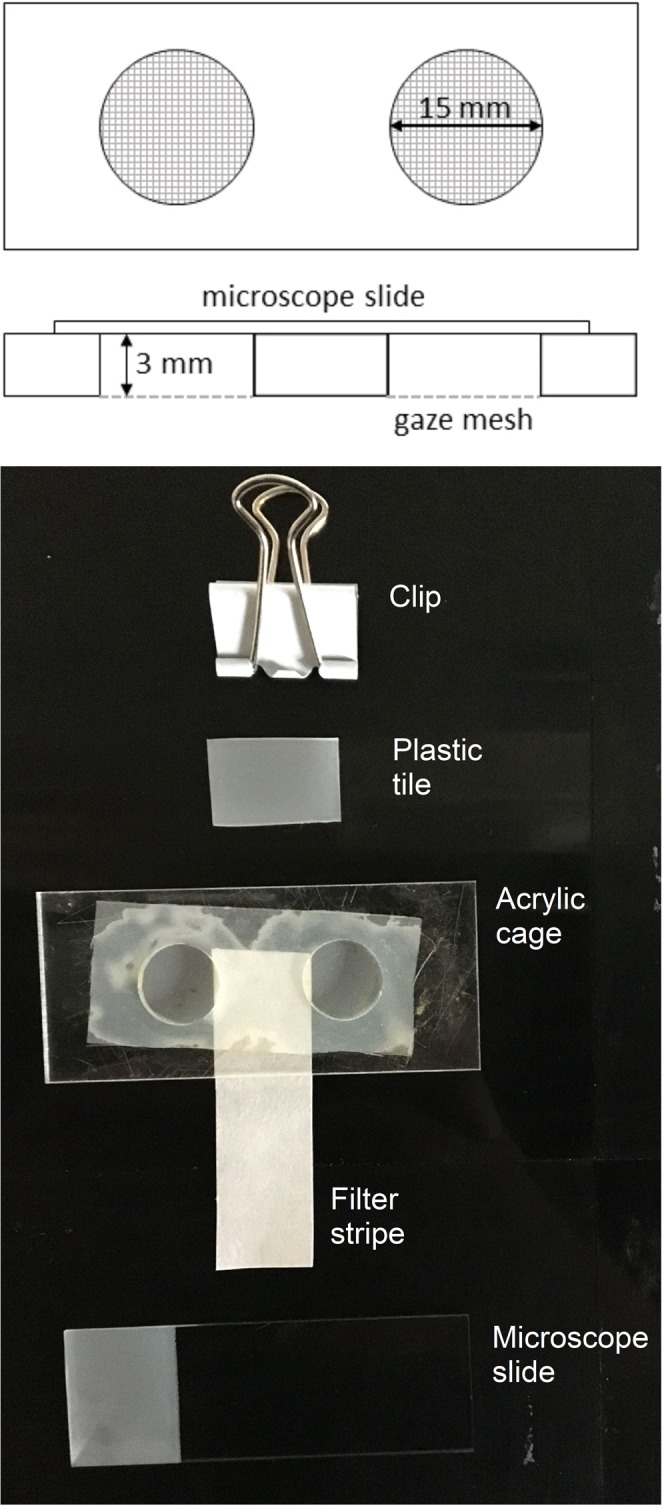
A lockable acrylic cage used as experimental unit from the top and lateral view. Single eggs of *A. limonicus* were placed in the cylindrical circular chamber (15 mm Ø, 3 mm height) and provided with spider mite eggs and pollen for food. A secured fine gaze mesh and a removable microscope slide is on the bottom and top side of the cage, respectively. Water supply was provided by a water-saturated filter stripe fixed with a plastic tile at the bottom of the cage. A clip is closing the cage.

Lockable acrylic cages were used as experimental units with cylindrical circular chambers (Ø 15 mm, 3 mm high). The backsides of the chambers were closed with fine gaze, the front sides were closed with a microscope slide fixed with metallic clips (Fig. 1). Water was provided by a wet filter paper fixed on the backside of the cage, reaching into a cup with tap water.

### Thermal effects during juvenile development of the F1 and F2 generation

Females from the lab population were placed in cohorts of 20 individuals on detached bean leaves provided with spider mite eggs and pollen, where they deposited eggs at constant 25 °C during 6 h. The mean age of the eggs (±3 h) corresponds to 7% of the embryonic development, which reduces potential confounding effects induced by the thermal environment of the females^15^. These F1 eggs were placed singly in lockable cages, provided with spider mite eggs, pollen and water, and exposed to summer conditions or heat wave conditions. The developmental progress of the juveniles from the F1 generation was observed in 8 h and 16 h intervals per day until the individuals died or reached adulthood.

In order to get eggs for the F2 generation, cohorts of 20 gravid F1 females, reared under summer or heat wave conditions, were allowed to reproduce on detached bean leaves over 6 h. These F2 eggs (±3 h old) from each of the two F1 groups were further split in two groups and exposed to summer conditions or heat wave conditions. The developmental progress of the juveniles from the F2 generation was observed twice per day until the individuals died or reached adulthood. The F1 rearing temperature (T_F1_) was matching the F2 rearing temperature (T_F2_), when (1) T_F1_ (summer conditions) = T_F2_ (summer conditions) and (2) T_F1_ (heat wave conditions) = T_F2_ (heat wave conditions) and not matching T_F2_, when (1) T_F1_ (summer conditions) ≠ T_F2_ (heat wave conditions) and (2) T_F1_ (heat wave conditions) ≠ T_F2_ (summer conditions). The F1 and F2 experiments were replicated 26 to 29 and 23 to 27 times, respectively.

After the completion of the experiments, all adults from the F1 and F2 generation were mounted in a drop of Hoyer’s medium^39^ for body size measurement and sex determination. The distances between the bases of eight setae, i.e. j3, s4, S4, Z5 at both sides of the dorsal shield were measured for the females and males (see^40^ for a detailed description of the setal nomenclature in phytoseiid mites). Then an approximation value for the dorsal shield perimeter was calculated, which corresponds to 85–90% of the total perimeter of the dorsal shield^41^.

### Statistical analyses

All statistical analyses were carried out using SPSS 24^42^. First, a chi-square test was used to evaluate the temperature effects on the number of males and females from the F1 generation. The sex-ratios of the F1 generations reared under summer conditions (13 males, 10 females) and heat wave conditions (12 males, 15 females) were not affected by the rearing temperatures (*P* = 0.395), so that sex could be used as independent factor in the statistical analyses of the F1 generation. Second, generalized linear models (GLMs) were applied to analyze the effects of temperature [F1 generation: summer or heat wave conditions; F2 generation: (1) F1 rearing temperatures (T_F1_): summer or heat wave conditions), (2) F2 rearing temperatures (T_F2_): summer or heat wave conditions)] during juvenile development on the survival proportions (binomial distribution, logit link function) of *A. limonicus* from the F1 and F2 generation. Third, multivariate analyses of variance (MANOVAs) with subsequent univariate analyses of variance (ANOVAs) were used to analyze (1) the effects of temperature (summer or heat wave conditions) and sex (female or male) during juvenile development on age and size at maturity of the F1 generation, and (2) the effects of T_F1_ (F1 rearing temperatures: summer or heat wave conditions) and T_F2_ (F2 rearing temperatures: summer or heat wave conditions) on age and size at maturity of the F2 generation for each sex separately. To detail differences on age and size at maturity of the thermal regimes between (F1 generation) and within (F2 generation) males and females, the data were compared by Fisher’s least significant difference (LSD) tests, if needed.

## Results

### Heat wave effects on the juvenile development of F1 generation

The survival probabilities of the F1 generation were high [summer conditions: 0.90 (proportion), heat wave conditions: 0.88)] and not affected by temperature (generalized linear model, GLM: X^2^_1_ = 0.034, P = 0.853).

**Figure 2:**
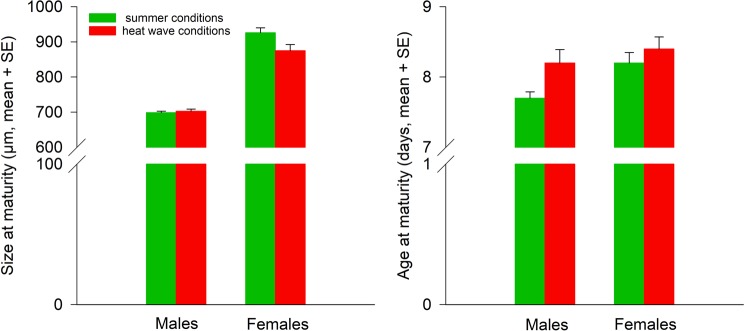
Temperature effects during juvenile development (summer conditions = green bars, heat wave conditions = red bars) on size and age at maturity of *Amblydromalus limonicus* males and females from the F1 generation.

Size at maturity of F1 *A. limonicus* was influenced by both main factors temperature and sex and their interaction (Table 2). Males were smaller than females (µm, mean ± SE; 701.18 ± 7.85 versus 900.91 ± 8.00). Adult body size was larger, when the juveniles were exposed to summer conditions (812.84 ± 7.59) than that juveniles were exposed to heat waves (789.25 ± 8.24). However, the temperature effects on body size at maturity were dependent on the sex. Female (pairwise LSD tests, summer versus heat wave conditions: P = 0.004), but not male body size (summer versus heat wave conditions: P = 0.800), decreased, when the juveniles were exposed to heat wave conditions (Fig. 2).

**Table 2:** Rearing temperature effects (summer conditions, heat wave conditions) and sex (females, males) on size and age at maturity of *Amblydromalus limonicus* from the F1 generation using univariate analyses of variance (ANOVAs).

Parameter	Source of variation	*F*	d.f.	P
Size	Temperature	4.074	1	0.049
	Sex	292.293	1	<0.001
	Temperature × sex	5.641	1	0.022
Age	Temperature	3.907	1	0.054
	Sex	6.363	1	0.015
	Temperature × sex	1.246	1	0.270

Results of the multivariate analysis of variance (MANOVA): temperature: Pillai’s trace = 0.129, d.f. = 2, P = 0.044; sex: Pillai’s trace = 0.875, d.f. = 2, P < 0.001; temperature × sex: Pillai’s trace = 0.148, d.f. = 2, P = 0.027).

Temperature and sex affected age at maturity of F1 *A. limonicus*, but not their interaction (Table 2). The juveniles exposed to summer conditions grew to adulthood earlier (days, mean ± SE; 7.99 ± 0.10) than those exposed to heat waves (8.31 ± 0.11). Males developed faster than females (7.95 ± 0.11 versus 8.35 ± 0.11) (Fig. 2).

### Heat wave effects on the juvenile development of F2 generation

Rearing temperature of the F1 generation (T_F1_) (generalized linear model, GLM: X^2^_1_ = 1.962, P = 0.161), rearing temperature of the F2 generation (T_F2_) (X^2^_1_ = 0.243, P = 0.622) and their interaction (X^2^_1_ = 0.292, P = 0.589) did not influence the survival proportions of *A. limonicus* from the F2 generation ranging from 0.83 to 0.96.

**Figure 3:**
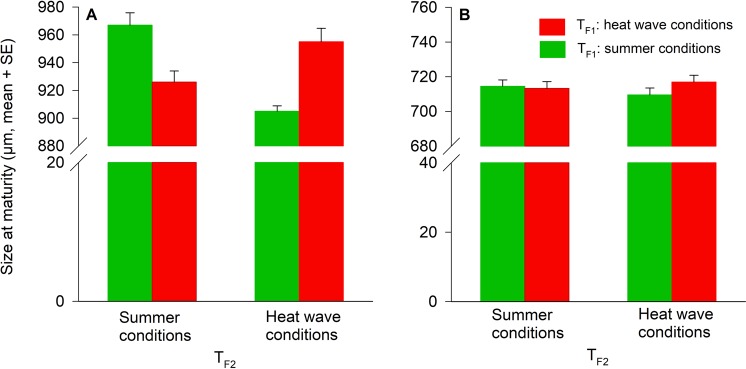
Temperature effects (T_F1_, T_F2_) during juvenile development on size at maturity of *A. limonicus* females (**A**) and males (**B**) from the F2 generation. T_F1_: Rearing temperature of the F1 generation (summer conditions, heat wave conditions). T_F2_: Rearing temperature of the F2 generation (summer conditions, heat wave conditions).

Body size at maturity of the F2 females was affected only by T_F1_ × T_F2_ (Table 3) indicating that the T_F2_ effects on female body size were dependent on T_F1_. F2 females were always larger, when their rearing temperatures (T_F2_) were matching T_F1_ (pairwise LSD tests, (1) T_F1_ and T_F2_ = summer conditions versus T_F1_ = heat wave conditions and T_F2_ = summer conditions: P > 0.001; (2) T_F1_ and T_F2_ = heat wave conditions versus T_F1_ = summer conditions and T_F2_ = heat wave conditions: P = 0.032) (Fig. 3). Size at maturity of the F2 males was not affected by T_F1_, T_F2_, and their interaction (Table 3, Fig. 3).

**Table 3:** T_F1_^1^ and T_F2_^2^ effects on size and age at maturity of *Amblydromalus limonicus* females and males from the F2 generation using univariate analyses of variance (ANOVAs).

Sex	Parameter	Source of variation	*F*	d.f.	P
Females	Size	T_F1_	0.299	1	0.587
		T_F1_ × T_F2_	27.312	1	<0.001
	Age	T_F1_	10.772	1	0.001
		T_F1_ × T_F2_	0.190	1	0.663
Males	Size	T_F1_	0.590	1	0.447
	Age	T_F1_	7.039	1	0.012

^1^T_F1_ = rearing temperature of the F1 generation (summer conditions or heat wave conditions).

^2^T_F2_ = rearing temperature of the F2 generation (summer conditions or heat wave conditions).

Results of the multivariate analyses of variance (MANOVAs): (1) females, T_F1_: Pillai’s trace = 0.188, d.f. = 2, P = 0.009, T_F2_: Pillai’s trace = 0.075, d.f. = 2, P = 0.175, T_F1_ × T_F2_: Pillai’s trace = 0.373, d.f. = 2, P < 0.001; (2) males, T_F1_: Pillai’s trace = 0.160, d.f. = 2, *P* = 0.043, T_F2_: Pillai’s trace = 0.055, d.f. = 2, *P* = 0.359, T_F1_ × T_F2_: Pillai’s trace = 0.031, d.f. = 2, *P* = 0.572).

**Figure 4:**
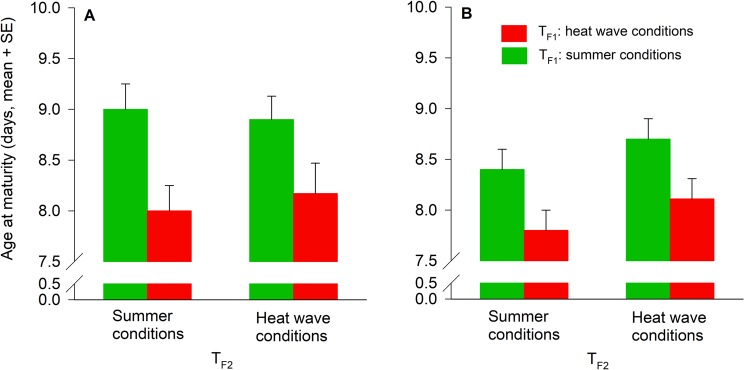
Temperature effects (T_F1_, T_F2_) during juvenile development on age at maturity of *A. limonicus* females (**A**) and males (**B**) from the F2 generation. T_F1_: Rearing temperature of the F1 generation (summer conditions, heat wave conditions). T_F2_: Rearing temperature of the F2 generation (summer conditions, heat wave conditions).

Age at maturity of the F2 females and males was only influenced by T_F1_ (Table 3). Females and males developed faster when deriving from the heat wave-experienced F1 generation (days, mean ± SE; females: 7.94 ± 0.17; males: 7.97 ± 0.15) compared to females and males deriving from the heat wave-naïve F1 generation (females: 8.10 ± 0.19; males: 8.56 ± 0.15) (Fig. 4).

## Discussion

Theoretically, the observed shifts in our experiments in relation to age and size at maturity in a terrestrial arthropod, the predatory mite *A. limonicus*, caused by temperature could be also affected by selective mortality, because these two traits (age at maturity: average heritability h^2^ ~0.26, body size at maturity: h^2^ ~0.4) are partially genetically determined^43^. However, independent of generation, survival was insensitive to heat waves, which clearly excludes the possibility of age- and size-specific survival of F1 and F2 individuals under heat wave conditions. Thus, our results provide a complex example of WGP and TGP modifications on fitness-relevant life history traits of *A. limonicus* as response to simulated heat waves. First, independent of generation, male body size was not affected by heat waves. Second, thermal WGP affected age at maturity of both F1 males and females, but only the F1 female size at maturity. Third, TGP effects influenced the age at maturity of males and females from the F2 generation. Finally, thermal TGP modifications on the body size of the F2 generation were sex-specific and affected only the F2 females.

The majority of juveniles from both F1 and F2 generation reached adulthood, when exposed to heat waves with daily T_max_ of 35 °C. In contrast, all *A. limonicus* eggs desiccated at constant temperatures and relative humidity of 35 °C and 60%, respectively^44^. Such different results related to the survival probabilities of terrestrial ectotherms confronted with constant or fluctuating heat stress during juvenile development were also documented for fruit flies^45^, aphids^46^, caterpillars^47^ and beetles^48^. Possibly, the daily recovery phases at moderate temperatures during heat wave exposure activated physiological adjustments such as the expression of heat-shock proteins and antioxidants^49^, which hindered the deathly consequences of overheating in *A. limonicus* eggs at constant temperatures. Thus, the evaluation of heat resistance at constant temperatures likely underestimates the survival probabilities of *A. limonicus* under natural conditions. This assumption is supported by the fact that the daily T_max_ values in summer often exceed 35 °C at the sampling location of the predatory mite in Northeastern Spain.

Most ectotherms respond to high temperatures by an accelerated development at the expense of smaller body size, which is termed the temperature-size rule^50,51^. Here, both males and females of the *A. limonicus* from the F1 generation reached later adulthood when exposed to heat waves. Provided that growth rates are not affected by environmental stress, prolonged development is usually correlated with larger body size^52^. In this study, male body size of *A. limonicus* was temperature-insensitive, but females reached smaller body sizes when reared under heat waves. Obviously, the thermal rearing conditions of the F1 generation also affected their growth rates following a general trend in arthropods: the long exposure to diurnal thermal fluctuations with high mean temperatures decreased the growth performance of the F1 generation under heat wave conditions^53^. The strength of the response, however, was sex-specific, because the magnitude of growth reduction was higher in females leading to smaller size.

Ultimately, WGP should come at high costs for the F1 generation reflected in reaching later adulthood under heat waves, which elongates time slot for predation and delays mating opportunities^14^. Additionally, there was a sex-specific cost for the heat wave-experienced females, which were also smaller than heat wave-naïve females. Female size is positively correlated with fitness-related traits such as longevity, mating success and fecundity in most arthropods^54,55^, including phytoseiid mites^24^. Thus, WGP responses to heat waves should have strong negative fitness-relevant effects on the F1 generation of *A. limonicus*, especially for the females.

Our results in relation to the juvenile F2 development may provide some evidence that two different types of TGP effects modified the F2 performance dependent on the affected trait (Bonduriansky & Crean 2017; Engqvist & Reinhold 2017). First, F2 males and females derived from the heat wave-experienced F1 generation reached earlier adulthood compared to offspring derived from the heat wave-naïve F1 generation. However, the change of age at maturity was only dependent on the F1 thermal conditions. Such TGP effects are termed condition-transfer effects, carry-over effects or silver-spoon effects, which are independent on the offspring environment^56–58^. Second, the change of size at maturity in the F2 females was both dependent on F1 and F2 thermal conditions. F2 females from the heat wave-experienced F1 generation were larger under heat wave conditions, but not summer conditions. Such TGP effects are termed anticipatory effects, when parents modify the phenotype of their offspring to the expected environment^56–58^.

One proximate explanation for these sex-specific results on the F2 body size might be the different thermal dependence of developmental and growth rates, which modulate the body size modifications^59^. The developmental rates were insensitive to heat stress in both females and males, so that only the growth rates of the F2 females could be affected by heat waves. Obviously, the direction of female growth rate modifications was dependent on the thermal environment of the F1 generation. High growth rates of F2 females from the heat wave-experienced F1 generation and low growth rates of F2 females from the heat wave-naïve F1 generation resulted in large and small daughterly body sizes, respectively.

Accelerated development may reduce the exposure time to heat waves. Thus, the F2 offspring should benefit from reaching earlier adulthood by trans-generational carry-over effects, when the adults are less heat-sensitive than the juvenile developmental stages. However, it remains an open question, if this is the case in predatory mites. Body size is an inverse function of temperature for the majority of ectotherms, but the potential benefits of being small at high temperatures remain elusive^60^. The most parsimonious explanation is that a moderate increase of mean temperatures signals higher ecosystem productivity, creating more favorable growing conditions for arthropods. Under such conditions it should pay off to invest in population expanding by speeding up development and starting early reproduction at the expense of smaller body size^50^. This benefit of being small at moderate high temperatures could be a cost at extreme high temperatures, because small individuals are highly vulnerable to dehydration and overheating^61,62^. Such a relationship between body size and heat resistance is documented both for vertebrates^63^ and arthropods^9,64,65^. Consequently, extreme high temperatures should favor individuals reaching larger size at maturity^66^, which is empirically supported by few studies indicating WGP responses to heat stress^9,60,67,68^. Based on these considerations, we assume that the TGP effects in *A. limonicus* should increase the female heat resistance by large size.

Heat waves should be ideal agents of trans-generational modifications in arthropods with specific traits for several reasons. First, arthropods that are wingless or have rudimentary developed wings are forced to stay put when exposed to heat waves making plastic responses very likely. Second, if the duration of a heat wave is longer than the generation time of a given arthropod species, then trans-generational effects would be favoured due to the thermal matching of F1 and F2 environment^69^. Interestingly, the dispersal abilities are constricted and the developmental rates are fast enough to reach adulthood in few days in species, for which trans-generational responses to extreme high temperature events were documented (the grain aphid *Sitobion avenae*^17,70^, the spider mite *Tetranychus urticae*^18^ and the study object, the predatory mite *A. limonicus*). Third, the irregular incidence of heat waves contributes to the temporal heterogeneity of the thermal environment, which should promote the evolution of different phenotypic optima within or across generations^71^. Fourth, the adaptive value of trans-generational effects also depends on the costs associated with such modifications^72,73^. Here, trans-generational modifications resulted in faster development and larger body size. High F1 costs should arise, when the females invest in larger egg sizes lowering their prospective reproductive success^55^. However, egg size is a function of female size in several arthropod taxa^54^ and the heat wave-experienced F1 females of *A. limonicus* were small. Thus, small females should not produce large eggs because of their morphological and physiological constraints compared to standard-sized females^24^. More likely, the thermal trans-generational modifications were based on the transmission of nutrients or hormones to the eggs^8^ or molecular mechanisms such as DNA methylation and histone modification^73^. The costs of these mechanisms are considered to be relatively low compared to egg size manipulation^74^. Additionally, the F1 investment in offspring size was only executed in daughters, which should also lower the costs. Finally, adaptive trans-generational effects should enhance both F1 and F2 fitness^72–74^. Here, the F1 costs were high indicating non-adaptive WGP effects induced by heat waves, which reduces the individual fitness of the F1 as documented also for other arthropods^75^. The fitness loss from reducing own growth should be compensated by increased fitness in the F2, which may indicate adaptive trans-generational effects (fast development, large size)^72^. Further investigations, however, are needed to evaluate in detail the importance of the observed trans-generational effects on F2 fitness of the predatory mite *A. limonicus* exposed to heat waves.

The paucity of studies investigating heat wave-induced TGP effects strongly limits the ability to deviate robust generalizations. Nonetheless, we assume that (1) heat waves have the potential to induce phenotypic plasticity within and across generations in fast developing, small arthropods with limited dispersal abilities; and (2) such modifications of fitness-relevant traits may allow species to counter the detrimental consequences of heat waves.
