# Author Correction: Evidence of trans-generational developmental modifications induced by simulated heat waves in an arthropod

**DOI:** 10.1038/s41598-020-63871-2

**Published:** 2020-04-21

**Authors:** A. Walzer, H. Formayer, M.-S. Tixier

**Affiliations:** 1 Division of Plant Protection, Department of Crop Sciences, University of Natural Resources and Life Sciences, 1180 Vienna, Austria; 2 Institute of Meteorology, Department of Water, Atmosphere and Environment, University of Natural Resources and Life Sciences, 1180 Vienna, Austria; 3 CBGP, Montpellier SupAgro, INRA, CIRAD, IRD, Univ. Montpellier, Campus International de Baillarguet, CS 30016, 34988 Montferrier-sur-Lez, cedex Montpellier, France

Correction to: *Scientific Reports* 10.1038/s41598-020-61040-z, published online 05 March 2020

A reference to the study describing the design of the cages was omitted in this Article. This paper is cited as Reference 1 below, and should appear in the text of the Article, in the Methods subsection 'Rearing and experimental units’, where,

“Lockable acrylic cages were used as experimental units with cylindrical circular chambers (Ø 15 mm, 3 mm high). The backsides of the chambers were closed with fine gaze, the front sides were closed with a microscope slide fixed with metallic clips (Fig. 1). Water was provided by a wet filter paper fixed on the backside of the cage, reaching into a cup with tap water.”

should read:

“Lockable acrylic cages were used as experimental units with cylindrical circular chambers (Ø 15 mm, 3 mm high). The backsides of the chambers were closed with fine gaze, the front sides were closed with a microscope slide fixed with metallic clips (Fig. 1). Water was provided by a wet filter paper fixed on the backside of the cage, reaching into a cup with tap water^1^.”
